# Professional Appointments

**Published:** 1849-09

**Authors:** 


*Professorial Appointments.*—Dr. Thomas Spencer, late Prof. of the Institutes and Practice of Medicine in Geneva Medical College, has been elected to the chair of Practice in the Rush Medical College, Chicago, Illinois, and has accepted the situation. Dr. N. S. Davis, late Editor of the Annalist, has been elected to the chair of Physiology in the same Institution, and has signified his acceptance.

